# Novel Effective Small-Molecule Antibacterials against *Enterococcus* Strains

**DOI:** 10.3390/molecules22122193

**Published:** 2017-12-10

**Authors:** Kerolos Ashraf, Kaveh Yasrebi, Tobias Hertlein, Knut Ohlsen, Michael Lalk, Andreas Hilgeroth

**Affiliations:** 1 Institute of Pharmacy, Martin-Luther-University Halle-Wittenberg, 06120 Halle, Germany; kerolos.ashraf@web.de (K.A.); kaveh.yasrebi@pharmazie.uni-halle.de (K.Y.); 2 Institute of Molecular Infection Biology, Julius-Maximilians-University Wuerzburg, 97080 Wuerzburg, Germany; tobias.hertlein@uni-wuerzburg.de (T.H.); knut.ohlsen@uni-wuerzburg.de (K.O.); 3 Institute of Biochemistry, Ernst-Moritz-Arndt-University Greifswald, 17489 Greifswald, Germany; lalk@uni-greifswald.de

**Keywords:** antibacterial activity, synthesis, derivatives, structure-activity, lead structure

## Abstract

*Enterococcus* species cause increasing numbers of infections in hospitals. They contribute to the increasing mortality rates, mostly in patients with comorbidities, who suffer from severe diseases. *Enterococcus* resistances against most antibiotics have been described, including novel antibiotics. Therefore, there is an ongoing demand for novel types of antibiotics that may overcome bacterial resistances. We discovered a novel class of antibiotics resulting from a simple one-pot reaction of indole and *o*-phthaldialdehyde. Differently substituted indolyl benzocarbazoles were yielded. Both the indole substitution and the positioning at the molecular scaffold influence the antibacterial activity towards the various strains of *Enterococcus* species with the highest relevance to nosocomial infections. Structure-activity relationships are discussed, and the first lead compounds were identified as also being effective in the case of a vancomycin resistance.

## 1. Introduction

Of the various *Enterococcus* species, *E. faecalis* and *E. faecium* are the most prevalent species in humans, making up more than 90% of *Enterococcus* species isolated in hospitals [1,2]. They cause infections that become most critical in patients with severe diseases like cancer, and additional bacterial infections [1,3]. In cases of such severe diseases, *Enterococcus* infections may contribute to increasing mortality rates [4,5].

Intrinsic and acquired *Enterococcus* resistances make antibiotic therapies difficult [1]. Intrinsic resistances against cephalosporins and penicillinase-resistant penicillins, as well as low-level resistance against aminoglycosides, strongly limit effective use of antibiotics in therapy [1]. Moreover, acquired resistances against tetracyclines, fluoroquinolones, aminoglycosides used in high doses, and vancomycin strengthen the critical outcome of such therapies [1]. Such resistances are often associated with transposons and are horizontally transferred by conjugative plasmids. Importantly, such transferred resistances have also been observed in cases of vancomycin between *Enterococcus* and *Staphylococcus* species, and contribute to an intensification of critical resistance development [6,7].

Newer antibiotics such as linezolid or daptomycin have been helpful in cases of known antibiotic resistance, but resistances to such novel drugs have also been described, resulting from mutations, enzymatic modification of the target structure, or active drug efflux [1,8]. Daptomycin resistances have been reported for both *E. faecalis* and *E. faecium* [9].

Therefore, there is a strong demand to find novel antibiotics that are not affected by bacterial resistance mechanisms against the known antibiotics.

The search for novel antibiotics is presently concentrated on natural sources like plants and marine fungi, sponges and cyanobacteria [10,11,12]. Recently discovered cyanobacterial metabolites, such as apratoxin A or largazole as cyclic depsipeptides, have shown some anticancer activities [13,14]. Cyanobacterial calothrixins A and B as indolophenanthridine compounds possess antiparasitic and antibacterial activities in addition to their cytotoxic properties, which may result from the quinone substructure within the molecular scaffold [13,15]. The isolated yields of those natural compounds are mostly low, so that complicated synthetic procedures are generally necessary to rebuild such structures in laboratories for later industrial use as potential drugs [13].

Calothrixins contain the indole subunit, which is found in many natural compounds with promising biological activities. We developed novel indolo compounds by a simple one-pot reaction of indole and *o*-phthaldialdehyde. The resulting indolyl benzocarbazoles were evaluated as antibacterial agents against the *Enterococcus* species *E. faecalis* and *E. faecium* with respect to nosocomial *Enterococcus* infections, showing promising activities of the identified lead structures.

## 2. Results and Discussion

### 2.1. Synthesis of the Indolyl Benzocarbazoles

**Scheme 1:**
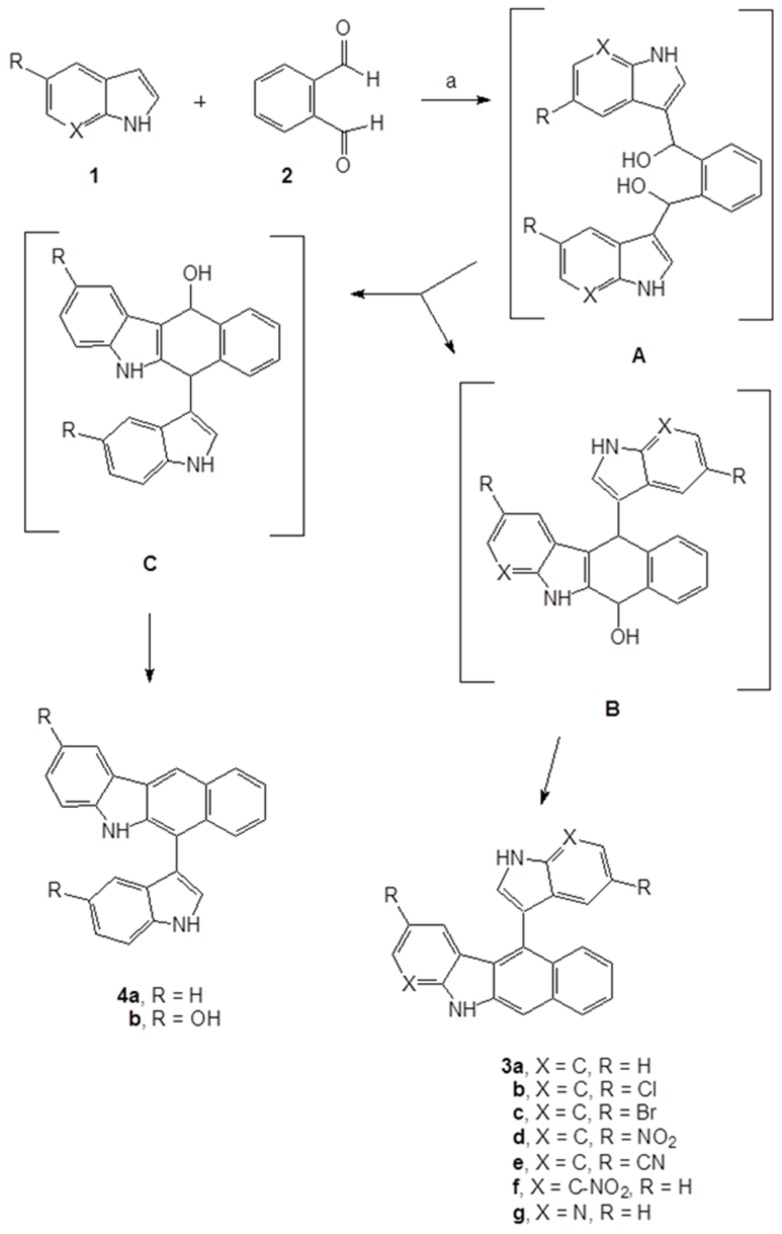
Synthesis of the indolyl benzocarbazole types **3** and **4**, (a) 100 °C, CH_3_COOH.

The synthesis of our indoyl benzocarbazoles started with 2 equivalents of indole and 1 equivalent of *o*-phthaldialdehyde (Scheme 1).

The 3-position of an unsubstituted indole is preferably attacked by electrophilic agents as an electron-rich position [16]. Under the acetic acid conditions used, both protonated carbonyl functions of the dialdehyde undergo an electrophilic attack at the electron-rich 3-position of the indole, giving the first reaction, and resulting in intermediate **A**. Next, one of the hydroxyl functions is protonated, and a water molecule elimination follows. The remaining carbenium ion attacks the 2-position of the neighboured indole function, giving either the second intermediate **B** with the indole substituent at the 11-position of the molecular scaffold after ring closure, or the third intermediate **C** with the indole substituent at the 6-position. In the case of 7-azaindole **1g**, we were able to isolate intermediate **B,** which supports the mechanism for the observed product formation. The compound’s characteristics were: C=N IR vibration band at 1687 cm^−1^; signal of the hydroxyl function in the NMR spectrum at 5.95 ppm; and the determined ESI mass with 335.7 as M + H^+^ peak [17]. In the case of the other indoles, the corresponding intermediate will have been formed, too. Finally, the hydroxyl functions in both intermediates **B** and **C** are protonated and a second water molecule elimination follows. Then, an aromatizing reaction leads to the differently substituted indolyl benzocarbazoles **3** and **4**. In the case of the unsubstituted indole starting compound, we found both indolyl benzocarbazole types **3a** and **4a**. The 5- and 7-substituted indoles with mostly electron withdrawing groups (EWG) resulted in the 11-indolyl substituted benzocarbazoles **3a**–**g**; whereas, in the electron-donating group (EDG), the 5-hydroxy function led to the 6-indolyl benzocarbazole **4b**. The NMR spectra of both indolyl benzocarbazole types were characterized by the two signals for the *N*H functions. In the case of compound **3a**, which was representative of the 11-indoyl benzocarbazole type **3**, the 6-proton signal appeared at 7.94 ppm; whereas for the 6-indolyl benzocarbazole type **4**, the signal of the representative compound **4a** appeared at 8.72 ppm. The low-field shift of the 6-proton resulted from the neighbored *N*H function, which was in accordance with predicted NMR data and proton shift data of indole protons next to the *N*H function of related carbazoles [18].

### 2.2. Antimicrobial Activity of the Indolyl Benzocarbazole Types **3** and **4**

The antibacterial activities of the compounds were characterized as minimal inhibitory concentration (MIC) values of growth determined by optical density. The activity was evaluated against various *Enterococcus* strains of *E. faecalis* and *E. faecium*, including one vancomycin-resistant *E. faecium* strain using the 2-fold serial dilution technique by starting with test compound stock solutions of 512 μg/mL.

The unsubstituted indolyl benzocarbazole compound **3a** showed only residual activities towards the tested *E. faecalis* strains (Table 1). The 5-chloro indole substitution of derivative **3b** significantly increased the activity towards one *E. faecalis* strain, with a MIC value of 20 μM. A replacement of the chloro with a bromo substituent in compound **3c** resulted in an almost unchanged activity compared to compound **3b**. The 5-nitro substitution in compound **3d** additionally increased the activity towards the other *E. faecalis* strain, with both MIC values being 19 μM. The 5-cyano substitution of derivative **3e** further increased the activity against one strain to 10 μM compared to compound **3d**, whereas that against the other one decreased to 42 μM.

A movement of the favorable nitro function from the 5- to the 7-position of the indole led to major decreases of activity for compound **3f** towards both strains, with merely residual activities similar to the indole-unsubstituted compound **3a**. The 7-aza function of compound **3g** increased the activity towards one *E. faecalis* strain to 48 μM.

The unsubstituted indolyl benzocarbazole **4a** with the indole residue at the 6-position showed significantly improved activities towards both *E. faecalis* strains with 24 μM and 12 μM, respectively. The 5-hydroxy indole substitution in compound **4b** led to a decreased activity in one *E. faecalis* strain, whereas the activity towards the other strain remained unchanged.

It can be concluded that 5-substitutions of the indole residue in compound type **3** with the indole residue at the 11-position increase the activity against *E. faecalis*. However, the best activities against both *E. faecalis* strains were observed for the unsubstituted indole derivative of compound type **4** with the indole residue at the 6-position of the molecular scaffold.

The activity of the unsubstituted 11-indolyl compound **3a** against the *E. faecium* strain was almost as good as that of the vancomycin comparison used, with a MIC value of 6 μM. The 5-chloro substitution in compound **3b** increased the activity to 2 μM. The 5-bromo substitution of compound **3c** resulted in a further strengthened activity with a MIC value of 1 μM. The 5-nitro compound **3d**, however, was no longer active, whereas the 5-cyano function of compound **3e** showed only slight decreases in activity, with a MIC value of 10 μM. The nitro function placed in the 7-indole position of compound **3f** led to unchanged activity compared to that of the 5-position. However, the 7-aza indole derivative **3g** showed activity with a MIC value of 24 μM. Both compounds of type **4** showed activities similar to the used vancomycin.

Finally, we determined the activity against a vancomycin-resistant *E. faecium* strain. The unsubstituted indole derivative **3a** resulted in a MIC value of 48 μM, whereas both the various 5- and 7-substituted indole derivatives **3b**–**g** all showed significantly decreased activities. The best activities against the resistant strain were determined for the unsubstituted indole derivative **4a** with the indole residue in the 6-position and its 4-hydroxy derivative **4b**, with 12 μM and 22 μM, respectively.

It can be concluded that, similar to the activities against *E. faecalis*, the observed 5-indole substitutions increase the activity of the indolyl benzocarbazoles **3** up to that of vancomycin, with the exception of the 5-nitro function, which was unfavorable, also in the 7-position. The 6-indolyl benzocarbazoles **4** showed the best activities against the vanomycin-resistant strain, and were almost as favorable as vancomycin against the non-resistant *E. faecium* strain. However, the susceptibility of the enterococcal strains is highly diverse, depending on the compound tested. If this is due to a specific resistance mechanism, or depends on a different target susceptibility, it will be investigated in future work.

## 3. Material and Methods

### 3.1. Chemical Reagents and Instruments

Commercial reagents (Sigma-Aldrich Chemistry GmbH, Munich, Germany) were used without further purification. The ^1^H-NMR spectra (400 MHz) were measured using tetramethylsilane as internal standard. TLC was performed on E. Merck 5554 silica gel plates (Merck KGaA, Darmstadt, Germany). The high-resolution mass spectra were recorded on a Finnigan LCQ Classic mass spectrometer (Thermo Fisher Scientific Inc., Berlin, Germany).

### 3.2. General Procedure for the Synthesis of Compounds **3a**–**g** and **4a**,**b**

Phthaldialdehyde (1 mmol) was dissolved in glacial acetic acid (15 mL). Then, the corresponding indole (2 mmol) was added to the solution. The mixture was stirred under reflux conditions at 100 °C until all of the starting phthaldialdehyde had disappeared, according to TLC-monitoring in CH_2_Cl_2_ (100%). The reaction time varied from 2 to 12 h. The reaction mixture was worked up by a dropwise neutralizing of the acid with NaOH (10%) until a pH of 7 was reached, followed extraction with CH_2_Cl_2_ (50 mL) for three times. The organic layer was washed with water and brine each for three times. Then it was dried over sodium sulphate, filtered and concentrated in vacuum. The reaction products were given after column chromatography using silica gel and ethyl acetate/cyclohexane mixtures as eluent. The resulting fractions (each 10 mL) were analyzed by TLC to identify the compound containing fractions which were unified and evaporated to dryness to give the target compounds.

*11-(1H-Indol-3-yl)-5H-benzo[b]carbazole* (**3a**). Yield 23%; m.p. 255–260 °C; IR (ATR) ν = 3407 (NH) cm^−1^; ^1^H-NMR (acetone-*d*_6_) δ = 10.47 (s, 1H, NH), 10.29 (s, 1H, NH), 8.02 (m, 1H), 7.94 (s, 1H, 6-H), 7.89 (m, 1H), 7.68 (dt, *J* = 8.3, 1.0 Hz, 1H), 7.57 (d, *J* = 2.3 Hz, 1H), 7.44 (dt, *J* = 8.1, 1.2 Hz, 2H), 7.26 (m, 3H), 7.06–6.89 (m, 3H), 6.75 (m, 1H); *m*/*z* (ESI) 333.2 (M + H^+^).

*2-Chloro-11-(5-chloro-1H-indol-3-yl)-5H-benzo[b]carbazole* (**3b**). Yield 37%; m.p. 230–235 °C; IR (ATR) ν = 3409 (NH) cm^−1^, 1609 (C–Cl); ^1^H-NMR (DMSO-*d*_6_) δ = 11.83 (s, 1H, NH), 11.45 (s, 1H, NH), 8.06 (m, 1H), 7.95 (s, 1H, 6-H), 7.69 (m, 3H), 7.45 (m, 2H), 7.34 (d, *J* = 7.6 Hz, 1H), 7.25 (m, 2H), 6.78 (d, *J* = 2.0 Hz, 1H), 6.72 (d, *J* = 2.1 Hz, 1H); *m*/*z* (ESI) 402.1 (M + H^+^).

*2-Bromo-11-(5-bromo-1H-indol-3-yl)-5H-benzo[b]carbazole* (**3c**). Yield 50%; m.p. 245–250 °C; IR (ATR) ν = 3408 (NH) cm^−1^, 1604 (C–Br); ^1^H-NMR (DMSO-*d*_6_) δ = 11.85 (s, 1H, NH), 11.46 (s, 1H, NH), 8.07 (m, 1H), 7.96 (s, 1H, 6-H), 7.70 (m, 2H), 7.60 (m, 1H), 7.47 (m, 3H), 7.30 (m, 2H), 6.93 (d, *J* = 1.9 Hz, 1H), 6.88 (d, *J* = 2.0 Hz, 1H); *m*/*z* (ESI) 491.2 (M + H^+^).

*2-Nitro-11-(5-nitro-1H-indol-3-yl)-5H-benzo[b]carbazole* (**3d**). Yield 20%; m.p. 140–150 °C; IR (ATR) ν = 3350 (NH) cm^−1^, 1324 (NO_2_); ^1^H-NMR (DMSO-*d*_6_) δ = 12.51 (s, 1H, NH), 12.23 (s, 1H, NH), 8.57 (d, 1H, *J* = 2.3 Hz, 1H), 8.26 (dd, *J* = 8.9, 2.3 Hz, 1H), 8.19 (dd, 1H, *J* = 8.4, 2.4 Hz, 1H), 8.16 (s, 1H), 8.13 (d, *J* = 8.4 Hz, 1H), 8.06 (d, *J* = 2.4 Hz, 1H), 7.98 (dt, *J* = 8.9, 2.4 Hz, 1H), 7.89 (d, *J* = 8.9 Hz, 1H), 7.77 (dd, *J* = 8.9, 2.4 Hz, 1H), 7.70 (d, 1H, *J* = 2.3 Hz), 7.56 (dt, *J* = 8.9, 1.8 Hz, 1H), 7.36 (dd, *J* = 8.9, 1.8 HZ, 1H); *m*/*z* (ESI) 423.1 (M + H^+^).

*11-(5-Cyano-1H-indol-3-yl)-5H-benzo[b]carbazole-2-carbonitrile* (**3e**). Yield 12%; m.p. 210–215 °C; IR (ATR) ν = 3311 (NH) cm^−1^, 2219 (CN); ^1^H-NMR (acetone-*d*_6_) δ = 11.43 (s, 1H, NH), 10.97 (s, 1H, NH), 8.10 (m, 2H), 7.92 (m, 2H), 7.84 (m, 1H), 7.70–7.60 (m, 2H), 7.60–7.54 (m, 2H), 7.43 (m, 2H), 7.19 (m, 1H); *m*/*z* (ESI) 383.6 (M + H^+^).

*4-Nitro-11-(7-nitro-1H-indol-3-yl)-5H-benzo[b]carbazole* (**3f**). Yield 55%; m.p. > 320 °C; IR (ATR) ν = 3400 (NH) cm^−1^, 1330 (NO_2_); ^1^H-NMR (DMSO-*d*_6_) δ = 12.47 (s, 1H, NH), 12.30 (s, 1H, NH), 10.46 (s, 1H), 8.26 (m, 1H), 8.14 (dd, *J* = 7.5, 1.5 Hz, 1H), 7.99 (dd, *J* = 7.4, 1.6 Hz, 1H), 7.85 (m, 1H), 7.70 (m, 1H), 7.55 (m, 1H), 7.37 (m, 2H), 7,10 (m, 2H), 6.97 (t, *J* = 7.5 Hz, 1H); *m*/*z* (ESI) 423.4 (M + H^+^).

*5-(1H-Pyrrolo[2,3-b]pyridine-3-yl)-11H-benzo[f]pyrido[2,3-b]indole* (**3g**). Yield 12%; m.p. > 320 °C; IR (ATR) ν = 3134 (NH), 1330 (C=N) cm^−1^; ^1^H-NMR (DMSO-*d*_6_) δ = 12.18 (s, 1H, NH), 11.83 (s, 1H, NH), 8.31 (m, 2H), 8.09 (m, 1H), 7.93 (s, 1H), 7.79 (m, 2H), 7.48 (ddd, *J* = 8.1, 6.6, 1.2 Hz, 1H), 7.29 (m, 2H), 7.05 (s, 1H), 6.98 (m, 1H), 6.84 (m, 1H); *m*/*z* (ESI) 335.7 (M + H^+^).

*6-(1H-Indol-3-yl)-5H-benzo[b]carbazole* (**4a**). Yield 4%; m.p. 115–120 °C; IR (ATR) ν = 3411 (NH) cm^−1^; ^1^H-NMR (acetone-*d*_6_) δ = 10.69 (s, 1H, NH), 9.77 (s, 1H, NH), 8.72 (s, 1H, 11-H), 8.31 (m, 1H), 8.15 (m, 1H), 7.91 (ddd, *J* = 8.3, 1.7, 0.8 Hz, 1H), 7.64 (m, 2H), 7.39 (m, 4H), 7.19 (m, 3H), 7.0 (ddd, *J* = 8.0, 6.9, 1.0 Hz, 1H); *m*/*z* (ESI) 333.1 (M + H^+^).

*11-(5-Hydroxy-1H-indol-3-yl)-5H-benzo[b]carbazole-2-ol* (**4b**). Yield 17%; m.p. 205–210 °C; IR (ATR) ν = 3402 (NH) cm^−1^; ^1^H-NMR (DMSO-*d*_6_) δ = 11.22 (s, 1H, NH), 10.06 (s, 1H, NH), 8.96 (s, 1H, 11-H), 8.56 (s, 1H, OH), 8.48 (s, 1H, OH), 8.10 (m, 1H), 7.76-7.23 (m, 7H), 6.89 (m, 1H), 6,66 (m, 1H), 6.34 (dd, *J* = 7.5, 1.5 Hz, 1H); *m*/*z* (ESI) 365.2 (M + H^+^).

### 3.3. Antimicrobial Activity

The compounds and the standard were dissolved in 12.5% DMSO at concentrations of 512 μg/mL. Further dilutions of the compounds and standard drug in the test medium were prepared at the required quantities of 256, 128, 64, 32, 16, 8, 4, 2, 1, 0.5 and 0.25 μg/mL concentrations with Mueller-Hinton broth. The minimum inhibitory concentrations (MIC) were determined using the 2-fold serial dilution technique. All the compounds were tested for their in vitro growth inhibitory activity against *Enterococcus faecalis* strains OG1X [19] and JH2-2 [20], *Enterococcus facium* strain 2121198 [21], and the vancomycin-resistant strain AW2 [22]. All strains were cultivated from the strain collection of the Institute of Molecular Infection Biology, University of Würzburg.

The cultures were obtained from Mueller-Hinton broth (Difco) for all the bacterial strains after 24 h of incubation at 37 ± 1 °C. Testing was carried out in Mueller-Hinton broth at pH 7.4, and the 2-fold serial dilution technique with Mueller-Hinton broth was applied using microtiter plates. Each test well was inoculated with 100 μL of the respective compound, and 100 μL bacterial suspension. The final inoculum size was 5 × 10^5^ CFU/mL for the antibacterial assay. A set of wells containing only inoculated broth was used as control. After incubation for 24 h at 37 ± 1 °C, the last well with no growth of microorganism was recorded to represent the MIC (expressed in μM). Two experimental and biological replicates were performed, with consistent results.

## 4. Conclusions

The finding of novel antibacterial agents is a great challenge today in combatting bacterial infections, which are difficult to defeat with the known antibiotics because of resistance developments, for various reasons. We discovered a novel class of antibacterial compounds that are accessible through a simple one-pot reaction, and thus may be produced without extensive costs for later use as antibiotics, in contrast to antibiotics from natural products. Within our compound class of differently substituted indolyl benzocarbazole compounds, we observed substituent-dependent effects of the evaluated antibacterial activity against the various *Enterococcus* species and strains. The observed 5-indole substituted compounds showed the most promising effects against almost all vancomycin-sensitive strains. Those with a 5-chloro and a 5-bromo substitution were identified as the lead compounds, with improved activities compared to vancomycin. Those 6-indolyl compounds, **4a**,**b**, proved to be active against the vancomycin-resistant *E. faecium* strain, and thus are perspective lead structures in similar cases of such antibiotic resistance.

## Figures and Tables

**Table 1:** Antibacterial activity of benzocarbazole types **3** and **4** against different *Enterococcus faecalis* and *E. faecium* strains.

MIC (μM)				
Compound	*E. faecalis* OG1X	*E. faecalis* JH2-2	*E. faecium* 2121198	*E. faecium* AW2VR ^1^
**3a**	386	386	6	48
**3b**	319	20	2	319
**3c**	261	16	1	261
**3d**	19	19	607	303
**3e**	42	10	10	166
**3f**	303	607	607	303
**3g**	48	767	24	383
**4a**	12	24	6	12
**4b**	11	44	5	22
Vancomycin	1	1	1	n.d. ^2^

^1^ VR = vancomycin-resistant; ^2^ n.d. = not determined.
